# Accounting for Imperfect Detection in Ecology: A Quantitative Review

**DOI:** 10.1371/journal.pone.0111436

**Published:** 2014-10-30

**Authors:** Kenneth F. Kellner, Robert K. Swihart

**Affiliations:** Department of Forestry and Natural Resources, Purdue University, West Lafayette, Indiana, United States of America; North Carolina State University, United States of America

## Abstract

Detection in studies of species abundance and distribution is often imperfect. Assuming perfect detection introduces bias into estimation that can weaken inference upon which understanding and policy are based. Despite availability of numerous methods designed to address this assumption, many refereed papers in ecology fail to account for non-detection error. We conducted a quantitative literature review of 537 ecological articles to measure the degree to which studies of different taxa, at various scales, and over time have accounted for imperfect detection. Overall, just 23% of articles accounted for imperfect detection. The probability that an article incorporated imperfect detection increased with time and varied among taxa studied; studies of vertebrates were more likely to incorporate imperfect detection. Among articles that reported detection probability, 70% contained per-survey estimates of detection that were less than 0.5. For articles in which constancy of detection was tested, 86% reported significant variation. We hope that our findings prompt more ecologists to consider carefully the detection process when designing studies and analyzing results, especially for sub-disciplines where incorporation of imperfect detection in study design and analysis so far has been lacking.

## Introduction

Measuring the abundance and distribution of organisms is a primary goal of ecology, conservation, and management [1]. These and related parameters (e.g. vital rates, species diversity) are essential to understanding population and metapopulation dynamics, community assembly, trophic interactions, conservation of threatened and endangered species, and the effects of management. Numerous advances in measuring these parameters have occurred over the past several decades, nearly all of which rely on counts and/or observations of organisms [2]. Unfortunately, in most cases complete counts (i.e., censuses) are impossible due to logistical constraints and the cryptic nature of many species [3]. Therefore, inference is based on a sample from the broader population of interest. Perfect or invariant detection is frequently assumed in count-based plant and animal studies [2]. Unfortunately, detection is rarely either perfect or constant due to observer error [4], species rarity [5] or because detection varies with confounding variables such as environmental conditions [6]. Regardless of its cause, we will refer to this condition henceforth as imperfect detection.

When detection is imperfect, additional steps are needed to improve inference. Failure to do so can result in biased estimation and erroneous conclusions. Numerous studies have demonstrated that detection varies among species, over time, and among habitats, and there may be serious consequences when this variability is ignored. For example, failure to correct for imperfect detection may result in bias in estimated relationships with ecological covariates [6], [7], estimates of species distribution or abundance that are inaccurate or mask trends [8]–[11], improper selection of indicator species [12], and misinterpreted components of fitness such as size-dependent survival and senescence [13]. These errors can misinform management and policy and erode trust in ecologists.

Authors of several landmark papers in the past century have (1) alerted the scientific community to the harmful effects of imperfect detection and (2) designed experimental and statistical approaches that explicitly incorporate detection probability. Petersen [14] and Lincoln [15] recognized the limitations of simple counts and proposed a basic method to account for imperfect detection in abundance estimation using capture histories of marked organisms. Their approach laid the foundation for future methods of estimating abundance and survival based on marked animals including the Cormack-Jolly-Seber model [16]–[18], the robust design model [19], [20], and numerous others with increasing complexity and ability to account for variation in detection [21]–[24]. Recent advances have merged analysis of animal movement with capture-recapture, resulting in spatial capture-recapture models to estimate density and other parameters of interest [25], [26]. When identifying individual organisms is infeasible, repeated counts [27] [4], [28] and distance sampling [29] may be useful to account for detection when estimating abundance, as long as within-sample double-counting of individuals is avoided.

Other population and community metrics (occurrence/occupancy, local colonization and extinction, richness, diversity, and turnover) can also be biased when imperfect detection is ignored [6], [30]–[32]. Methods exist to estimate these parameters while accounting for detectability, if data are collected in a way that allows the detection process to be modeled [1], [11], [31], [33]–[35]. This is often achieved through replicate surveys of sampling sites [31], [33], [34], [36] although other methods such as the collection of times to detection are also possible [37], [38]. Software packages have been developed to make the modeling advances described above more accessible to ecologists, including programs CAPTURE [39], MARK [40], DISTANCE [41], PRESENCE [42], COMDYN [43], and E-Surge [44], as well as numerous R packages including ‘marked’ [45] ‘unmarked’ [46] and ‘secr’ [47]. Recently, Bayesian modeling approaches have been introduced to ecologists as an alternative means of fitting complex population and community models [1], [26], [48]–[51].

Despite numerous articles alerting ecologists to the consequences of imperfect detection, and despite available models and software capable of addressing imperfect detection, we commonly encounter refereed publications that fail to acknowledge or account for the presence of non-detection error. These casual observations beg the question: how prevalent are methods that incorporate imperfect detection in the ecological literature, and how does this differ among various types of studies and over time? Identifying these patterns is crucial to understand barriers to incorporation of imperfect detection and to target areas in which ecological inference might be improved.

We posited that the expanding availability of analysis methods, software, and computing power has increased the probability that, over time, researchers account for imperfect detection. We suspected that adoption has varied among areas of ecological inquiry (e.g. [10], [52]), so we asked whether the probability of accounting for imperfect detection has varied with the type of organism studied, the level of biological organization studied, or the spatial scale studied. Because of the taxonomic focus of many of the researchers who have developed methods for addressing imperfect detection, we predicted that a greater proportion of studies focused on fish, mammals, and birds would incorporate imperfect detection than other groups (e.g. plants, invertebrates); however we expected this difference to have declined over time. We also predicted that studies conducted at higher levels of organization (e.g. communities vs. individual animals) and with greater spatial extents (e.g. regional or landscape vs. local) would be less likely to incorporate imperfect detection due to the difficulties of implementing more complex study designs with limited resources. To test our predictions, we conducted a quantitative review of ecological literature spanning 40 years, 6 taxonomic groups, 5 commonly reported parameters, 2 levels of biological organization, and 3 spatial scales to determine how the use of statistical methods that incorporate imperfect detection has varied among these variables and over time.

## Materials and Methods

### Article Selection

**Figure 1:**
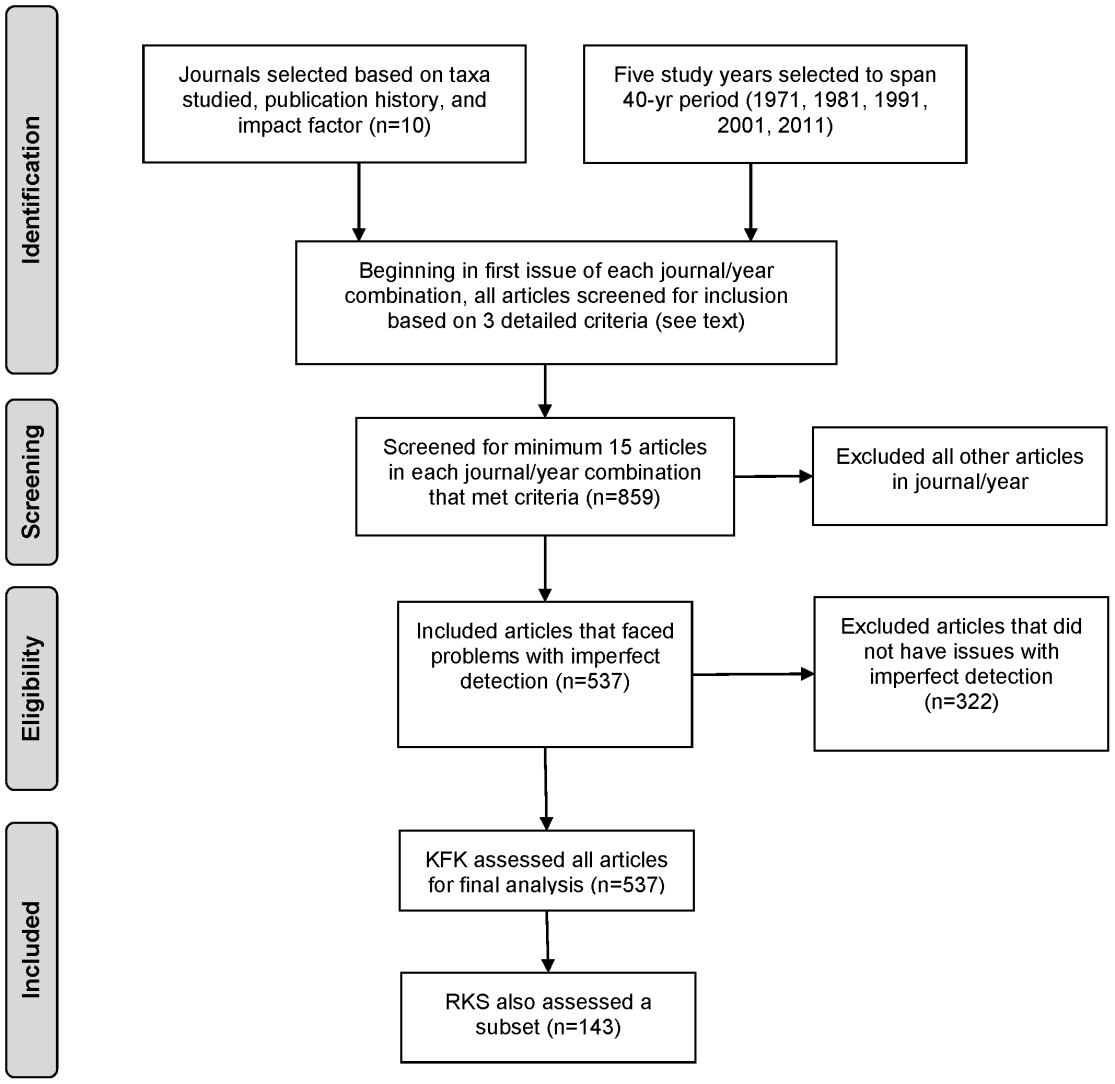
PRISMA flow diagram detailing how articles were selected for inclusion in the quantitative literature review.

A census of the literature was impractical, so we adopted a stratified sampling approach (Figure 1). We selected a subset of 10 journals to include in the study, chosen for their impact factors, long publication history, and coverage of a range of taxonomic groups (birds, fish, mammals, herpetofauna, invertebrates, and plants; Table 1). For each of the journals, we selected 5 years from which to sample papers: 1971, 1981, 1991, 2001, and 2011.

**Table 1:** Selected characteristics of the 10 journals included in a quantitative review examining use of methods that incorporate imperfect detection in wildlife and plant studies.

Journal Name	Taxa	Years Published	Impact Factor
Journal of Ecology	Plants	1913-	5.04
Journal of Animal Ecology	Multiple	1932-	4.94
Ecology	Multiple	1920-	4.85
Journal of Biogeography	Multiple	1974-	4.54
Ecological Entomology	Invertebrates	1836-	1.95
The Auk	Birds	1884-	1.81
Journal of Mammalogy	Mammals	1919-	1.74
Transactions of the American Fisheries Society	Fish	1872-	1.55
Journal of Wildlife Management	Multiple	1937-	1.36
Herpetologica	Reptiles, Amphibians	1936-	1.08

Journals were chosen for inclusion in the review on the basis of taxonomic groups studied, years published, and impact factor (from Journal Citation Reports 2011–2012).

Within each journal/year combination, we examined all articles (starting in the first issue), identifying those that fit our criteria for inclusion through careful reading of the abstract, methods, and results. Three criteria were defined: (1) the study had to be focused on one or more of the target taxonomic groups; (2) the study had to measure one or more of a set of parameters of interest, composed of abundance, occurrence, survival, richness or diversity, and extent or size of species range (selected because they are among those most often studied by ecologists and susceptible to the biases of imperfect detection); (3) the study had to be implemented in a way in which detection was likely imperfect, i.e., there was no evidence to support the feasibility of a complete census or to ensure constancy of detection. If fewer than 15 articles were selected in a given journal/year combination, we continued our search into the following year(s) until we had a minimum of 15 articles that fit the criteria.

### Data Collection

We recognized that our ability to correctly judge whether a study incorporated imperfect detection was likely less than perfect itself. We therefore implemented a replicated sampling approach commonly used as a means of parameter estimation when detection is imperfect. The objectives, methods, results, and discussion of each article were carefully read by the first author (KFK). The journal type (single-taxon focus vs. broader focus) year, taxa and parameters measured, experimental scale (population or community level), and spatial extent (local or landscape) were recorded for each article, as well as the presence/absence of an approach to account for imperfect detection. For the binary response variable, the presence of an approach included a statistical method of estimating detectability or an acknowledgment by the authors of imperfect detection as a potential issue. Articles that accounted for imperfect detection were further examined to determine (1) if they explicitly reported detection probabilities and (2) if and how detection varied (e.g. over time or among species). The second author (RKS) independently read and scored a subset of all articles in the same manner. Multiple “sampling occasions” for this subset of articles allowed for estimation of a detection parameter, analogous to a repeated-sample design for occupancy estimation [1], [31]. The approach we used assumes that there are no “false positives”, i.e., studies identified as accounting for imperfect detection when in truth they did not. We took a conservative approach to identifying studies that accounted for imperfect detection and believe false positives were negligible. Information about all articles included in the quantitative review is contained in the Dataset S1.

### Analysis

Using this repeated-sample data, we simultaneously modeled the probability that an article incorporated imperfect detection (hereafter *p*IID) and the probability that we were able to detect this when reading the articles (hereafter *p*R) using a hierarchical logistic regression model [1]. Year of publication, journal type, taxon and parameter(s) measured, scale, and spatial extent were considered as covariates on *p*IID. Taxon and reader (KFK or RKS) were considered as covariates on *p*R. In addition to the full model, we fit separate models for each taxon; these models did not have taxa as predictor variables but were otherwise identical to the full model. We explored models with interacting effects; however, finding no significant interactions, our final models included only additive effects. Models were fit in a Bayesian framework using JAGS [53], called from R [54] using the package R2jags [55]. We used two methods for assessing the statistical importance of each covariate in the models. First, we calculated a 95% credible interval for each parameter estimate based on the posterior distribution. Second, we calculated a parameter *f* representing the fraction of the sampled posterior distribution with the same sign as the mean; this value reflects our level of certainty that the parameter estimate is positive or negative.

## Results

A total of 537 articles surveyed by KFK fit the criteria and were included in the review. A subset (n = 143, 27%) was also examined by RKS. The most common parameters estimated were abundance (n = 377 articles), occupancy/occurrence (n = 121) and richness (n = 103), whereas survival (n = 66) and species range/distribution (n = 31) were less common. Birds (n = 154), invertebrates (n = 150), and mammals (n = 131) were the most commonly studied taxa.

**Figure 2:**
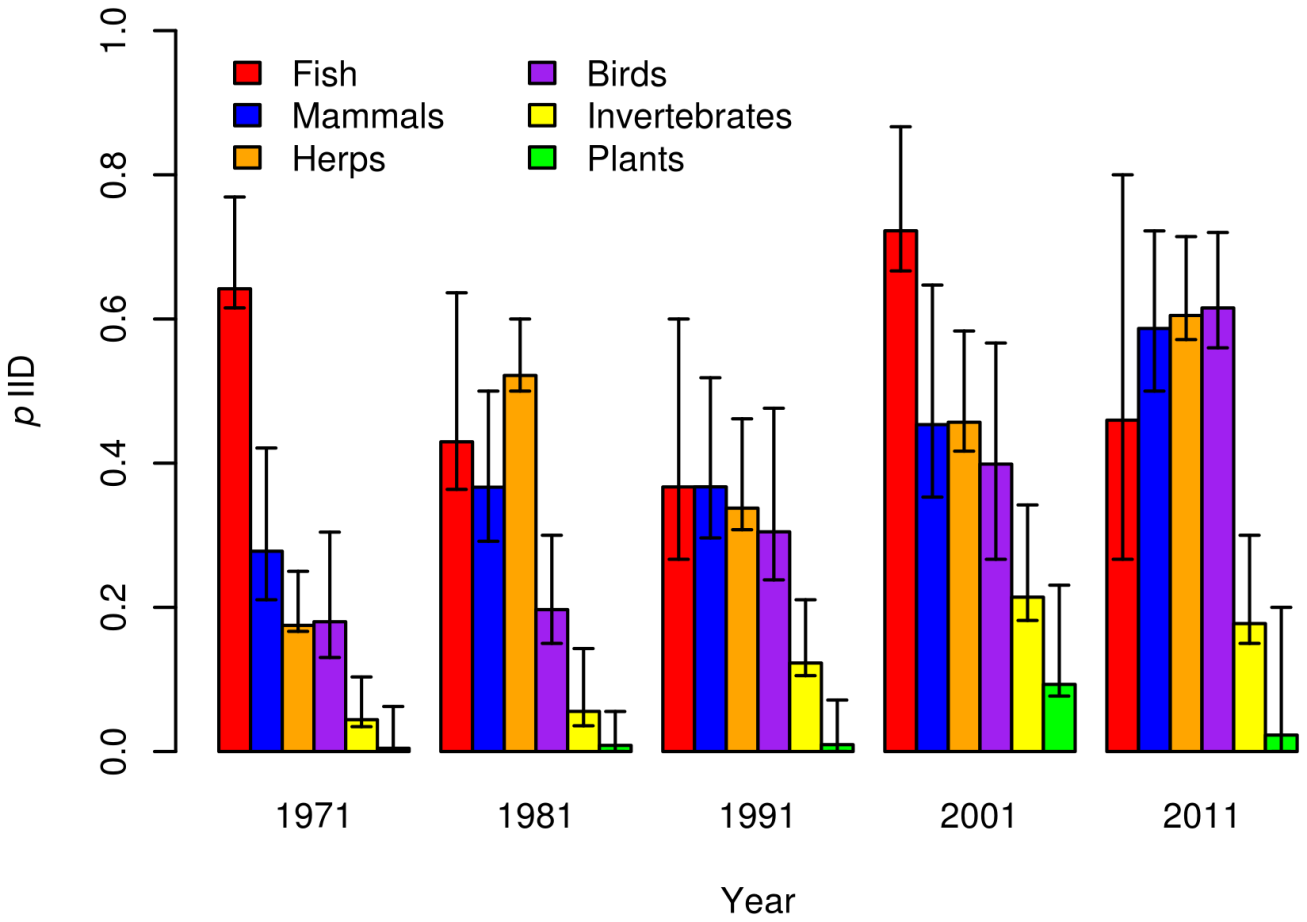
Mean estimated probability (p*IID*) that an article of a given taxon and year incorporated statistical methods to account for imperfect detection, based on output from the hierarchical logistic regression model. Error bars represent 95% credible intervals around the mean.

Overall, 23%±1.8 (123/537, mean±standard error) of articles addressed imperfect detection. There was a positive effect of year on *p*IID (*f* = 0.99, Table 2): the yearly mean percent of articles that addressed imperfect detection generally increased from 25%±5.9, 14%±4.0, and 23%±4.6 in 1971, 1981, and 1991, respectively, to 29%±5.1 and 35%±5.1 in 2001 and 2011. The same increasing trend over time appeared when taxa were modeled separately, with the exception of fish and plant articles (Figure 2, Table 3). Taxonomic group generally was an important covariate on *p*IID. Specifically, studies of fish were more likely (*f* = 0.97) to account for imperfect detection than the average (43%±5.9 of articles for fish vs. 23%±1.8 overall). In contrast, articles focused on plants (*f* = 0.99) and invertebrates (*f* = 0.95) were less likely to do so (1.4%±1.3 and 9.0%±2.7, respectively; Table 2). The remaining taxonomic groups (mammals, herpetofauna, and birds) were positively related to *p*IID, but with a lesser degree of certainty (Table 2). Articles in journals that focused on a single taxon were less likely (*f* = 0.98) to incorporate imperfect detection than multi-taxa journals.

**Table 2:** Estimated parameter values from a hierarchical logistic regression relating covariates (taxa, parameter estimated, year, journal type, and experimental scale) on the probability a given study accounted for imperfect detection.

Parameter	Estimate	95% Credible Interval	*f* b
Year	**0.45** a	(0.23, 0.66)	0.99
Fish	**1.01**	(0.01, 2.13)	0.97
Mammals	0.38	(−0.51, 1.49)	0.75
Herps	0.53	(−0.38, 1.50)	0.85
Birds	0.33	(−0.70, 1.22)	0.76
Invertebrates	–0.83	(−1.83, 0.16)	0.95
Plants	**–1.70**	(−2.95, −0.35)	0.99
Abundance	0.49	(−0.26, 1.18)	0.90
Occurrence	–0.60	(−1.42, 0.14)	0.95
Survival	**0.91**	(0.23, 1.65)	1.00
Richness	**–1.10**	(−1.81, −0.25)	0.99
Range	–0.92	(−2.17, 0.22)	0.94
Scale (1 = community)	–0.51	(−1.10, 0.08)	0.95
Spatial extent (1 = landscape)	–0.12	(−0.69, 0.45)	0.67
Journal type (1 = single taxon)	**–0.60**	(−1.12, −0.03)	0.98
*Detection Covariates*			
Observer (1 = RKS)	**1.33**	(0.16, 2.51)	0.98
Fish	–0.02	(−1.37, 1.51)	0.52
Mammals	–0.68	(−2.02, 0.52)	0.85
Herps	0.83	(−0.52, 2.36)	0.85
Birds	–0.62	(−1.80, 0.64)	0.84
Invertebrates	–0.14	(−1.70, 1.48)	0.57
Plants	–0.21	(−2.35, 1.77)	0.55

a Bolded parameters had 95% credible intervals that did not overlap 0 and are considered statistically important.

b Proportion of the parameter’s posterior distribution with same sign as the mean value; values approaching 1 reflect increasing certainty that the parameter is different from 0.

**Table 3:** Estimated parameter values (with 95% credible intervals in parentheses) from a series of hierarchical logistic regressions (separated by taxa) relating covariates (parameter estimated, year, journal type, and experimental scale) on the probability a given study accounted for imperfect detection.

	Taxon					
Parameter	Fish	Mammals	Herps	Birds	Inverts	Plants
Year	–0.19 (−0.66, 0.22)	**0.60** a (0.12, 1.16)	**0.44** (0.02, 1.01)	**0.79** (0.32, 1.30)	**0.54** (0.08, 1.07)	0.18 (−1.00, 1.68)
Abundance	0.51 (–0.83, 1.95)	**1.31** (0.09, 2.64)	0.38 ((–0.82, 1.69)	0.55 (–0.76, 2.00)	–0.67 (–1.99, 0.62)	0.08 (–1.84, 1.95)
Occurrence	–0.34 (–1.83, 1.20)	–0.20 (–1.62, 1.22)	–0.11 (–1.44, 1.21)	–1.05 (–2.38, 0.18)	–0.99 (–2.43, 0.27)	–0.15 (–1.89, 1.68)
Survival	1.17 (–0.34, 2.63)	0.09 (–1.23, 1.37)	0.55 (–0.84, 1.80)	**1.33** (0.09, 2.74)	1.09 (–0.38, 2.49)	–0.06 (–1.92, 1.81)
Richness	–0.58 (–2.08, 0.92)	–0.42 (–1.87, 1.22)	–0.98 (–2.72, 0.49)	–1.05 (–2.33, 0.11)	–0.16 (–1.31, 1.13)	–0.18 (–2.20, 1.69)
Range	–0.53 (–2.36, 1.17)	–0.53 (–2.35, 1.17)	–0.77 (–2.41, 0.85)	0.23 (–1.93, 0.15)	–0.45 (–2.17, 1.38)	–0.07 (–1.90, 1.69)
Scale (1 = community)	0.22 (–0.79, 1.27)	–0.55 (–1.60, 0.45)	–0.25 (–1.51, 1.08)	**1.27** (–2.46, −0.16)	–0.68 (–1.95, 0.61)	–0.52 (–2.51, 1.44)
Spatial extent (1 = landscape)	0.44 (–0.69, 1.65)	–0.77 (–1.98, 0.43)	–0.62 (–1.89, 0.46)	0.05 (–1.16, 1.30)	–0.10 (–1.36, 1.13)	–0.40 (–2.29, 1.65)
Journal type (1 = single taxon)	0.08 (–1.08, 1.14)	–0.78 (–1.79, 0.34)	0.05 (–1.20, 1.27)	–0.79 (–1.93, 0.15)	–0.03 (–1.18, 1.27)	0.12 (–1.75, 2.02)
*Detection covariates*						
Observer (1 = RKS)	0.64 (–1.15, 1.22)	0.01 (–2.22, 1.79)	0.23 (–1.43, 1.89)	0.01 (–2.13, 2.16)	0.08 (–1.83, 1.99)	–0.12 (–1.99, 1.62)

a Bolded parameters had 95% credible intervals that did not overlap 0 and are considered statistically important.

The parameter(s) estimated in each article also correlated with *p*IID. Articles that measured survival were more likely (*f* = 1.00) to incorporate imperfect detection than the mean (50%±6.1 vs. 23%±1.8); abundance was also positively related to *p*IID (Table 2). In contrast, papers that measured richness, occurrence/occupancy, and range/distribution were less likely to incorporate imperfect detection (6.0%±2.3, 10%±2.8, and 3.2%±3.2; Table 2). Articles examining entire communities were less likely to incorporate imperfect detection than studies focused on a single species (*f* = 0.95, Table 2). Spatial scale, in contrast, did not affect *p*IID (*f = *0.67, Table 2). The effects of parameters and scale on *p*IID were similar when taxonomic groups were modeled separately, with the exception of a positive effect of community studies on *p*IID for bird studies (Table 3).

The parameter *p*R did not differ between taxonomic groups. There was an observer effect, but it was small (Table 2). Just 10 of 143 papers (7%±2.1) examined by both readers had different assessments for the inclusion of imperfect detection.

Among articles that accounted for imperfect detection, 62 (50%) also reported information about detection probabilities in some form. Of these, 48%, 33% and 19% reported *maximum* estimated single-survey detection probabilities (i.e., probability of detection on a single sampling occasion) less than 0.7, 0.5, and 0.3, respectively. Most papers reported at least one detection probability significantly less than 1; 70% had at least one estimated detection probability less than 0.5, and 50% had at least one less than 0.3. Reported detection probabilities varied with at least one covariate in 86% of papers that tested for heterogeneity; the most common covariates were time (44%), species (25%), site or population (19%), and methodology (e.g. effort, observer effects; 19%).

## Discussion

### Taxonomic Groups

Our examination of ecology papers across 5 decades, 10 journals, 5 taxa, and other parameters of interest confirmed our initial observations: the majority (77%) of ecological studies failed to acknowledge or correct for imperfect detection when doing so likely would have been appropriate. As we predicted, there was considerable contrast among taxa: vertebrates (fish, mammals, birds, reptiles and amphibians) were more likely to account for imperfect detection than other taxa (plants and invertebrates). The earliest proponents of accounting for imperfect detection (e.g. [14], [15]) focused on vertebrates. The generally lower overall abundance, greater movement capability, and cryptic nature of vertebrates, relative to plants and invertebrates, may have encouraged earlier adoption by vertebrate ecologists of study designs and statistical methods to correct for limitations of sampling and detection. Many of these methods were published in taxon-specific journals or tailored to specific taxa (e.g. removal sampling of fish or mark-release-recapture of mammals and birds), so their adaptation to studies of other taxa was likely limited. Interestingly, single-taxon journals actually had a lower proportion of studies incorporate imperfect detection, likely reflecting the small number of studies in plant- and invertebrate-focused journals that did so.

The proportion of studies that accounted for imperfect detection increased over the 40-year period of our review for all vertebrate taxa except fish, which had a comparatively high proportion throughout (Figure 2). This positive trend presumably reflects greater awareness among vertebrate ecologists of the risks associated with failing to account for imperfect detection and a correspondingly greater availability of sampling and statistical methods designed specifically to account for errors of omission. While we did not collected detailed data on the specific statistical method(s) used to account for imperfect detection, we observed a corresponding generally positive trend in the diversity of statistical approaches used over time. Early studies (prior to 1981) of fish and mammals primarily used simple mark-release-recapture methods like the Lincoln-Peterson index [14], [15]. From 1981–2001 more complex mark-release-recapture methods [16]–[18] were most commonly used, but methods were still focused on specific taxa (primarily mammals and fish). From 2001 onward, the diversity of approaches greatly increased for most taxa thanks to the widespread introduction of occupancy modeling and related hierarchical modeling approaches applicable to wide variety of species [1], [31], [33], [36].

While the trend over time in adoption of methods that incorporate imperfect detection is less pronounced for plants and invertebrates (Figure 2), it is nevertheless positive and likely will continue as publications on these taxa draw attention to the issue [3], [56]–[59]. For example, [10] estimated detection probability for plants surveyed in the Swiss Biodiversity Monitoring program. Based on a random sample of 100 species (of 1700 detected), median single-survey detection probability was 0.74 (range 0.03–0.99) for the spring survey and 0.82 (range 0.03–0.99) for the late summer survey. Thus, distribution maps based on a single survey risk modeling the joint patterns of occurrence and detection. These two parameters potentially could be disentangled if they rely on covariates that are not identical and sample sizes are reasonably large [60], but these constraints suggest that researchers should use caution in making inferences from a single survey.

### Parameters Estimated

Each parameter measured in ecological studies presents unique logistical and statistical challenges. For example, estimating survival inherently requires marking individuals and following them through time, with collected data likely including multiple sampling events. Correcting for imperfect detection is a logical extension when these data are available, and the majority of survival studies did so. Important exceptions include survival studies that do not correct for conflation of non-detection with mortality, in which case an individual that is not detected at a given sampling occasion is assumed to be dead. The relatively high proportion of abundance studies that accounted for imperfect detection likely reflects a long history and proliferation of approaches to account for undetected individuals in abundance estimates [2], [61]. A notable exception to this pattern is the widespread use of “catch per unit effort” (CPUE) and similar metrics as indices of abundance. CPUE does not require marked organisms but implicitly assumes that detectability does not vary across time or experimental site, which has been shown to introduce bias into estimation [22], [62], [63].

Richness, occurrence, and range are closely related parameters. Richness can be represented as the sum of all species’ occurrences at a site, and range loosely corresponds to species occurrence across a large geographic area. They shared an additional characteristic in our study – a low probability of incorporating imperfect detection. Estimating these parameters (especially at the community scale) can require sampling for many species at numerous sites, requiring potentially difficult or costly repeated samples and/or careful allocation of sampling effort to obtain the data necessary to estimate detection. In addition, sampling designs and statistical methods that incorporate imperfect detection into estimation of occupancy and richness have appeared in the ecological literature only recently [31], [33], [34], [36]. For estimates of species range and distribution, a further issue is that researchers often have relied on historical presence-only data (e.g. from museum collections) which makes estimation of occupancy probability and subsequent inferences difficult [64] but not impossible [65]–[67], at least in a relative sense [68].

### Implications

We concede that it is impossible to know the extent of bias in past studies that have ignored imperfect detection. It certainly is possible that bias may have been small in some of the studies that failed to incorporate detection error, because detection probability was either high or invariant. However, for the subset of papers in our study that estimated detection probability, the median values of minimum and maximum single-survey detectability were 0.29 and 0.71, respectively, and 70% had at least one estimated detection probability less than 0.5, an indication that detection can commonly be much less than perfect. Admittedly, these studies are not a random subset of the studies we considered, as their authors presumably considered imperfect detection to be problematic in their study system. Still, the magnitude and frequency of bias seen in these and other studies (e.g. [6]–[13]) suggests strongly that imperfect detection is common and can weaken inference for many types of ecological processes. For example, simulations have demonstrated that ignoring imperfect detection, as is done with presence-absence and presence-only data, can dramatically diminish a model’s capacity to identify environmental correlates of species distributions [32].

Even if detection is imperfect, bias may not affect inference adversely for some purposes if detection is invariant. For instance, population indices are commonly used to monitor trends and assume constant detectability relative to abundance [69], [70], even though evidence of variation is common [71] and led [72] to recommend that the burden of proof should be shifted to demonstrate that detection probability is invariant. Our results provide support and warrant expansion of their recommendation; in 86% of papers from our study that examined the constancy assumption, detectability varied significantly. Rather than assume that detection is perfect, it seems prudent, then, for field ecologists to assume that detection probabilities differ and to require evidence of their equivalence before using indices or other measures that conflate this nuisance parameter with the parameter(s) of ecological interest.

Collectively, our findings should concern ecologists in search of stronger inference, and particularly managers and policy makers whose decisions often depend on accurate knowledge of species presence or abundance. Accounting for imperfect detection has been the exception and not the rule in ecology across most sub-disciplines and study types. We hope the patterns we have identified will encourage ecologists to consider carefully the detection process when designing studies and analyzing results. This is particularly important with taxonomic groups and parameters we highlighted that, in the past, have generally not accounted for imperfect detection.

We recognize that the methods developed thus far to deal with imperfect detection are not a panacea for estimation. For instance, methods to address imperfect detection, as with all estimation approaches, rely on assumptions that need to be carefully evaluated and may not be suitable for certain species or systems (e.g., [73], [74]). Further, addressing imperfect detection will not solve other forms of hidden bias that can afflict observational studies [75]. But we disagree with the suggestion that ignoring imperfect detection may be preferable to accounting and adjusting for it [76], [77]. We believe that a more suitable approach to improved estimation is to minimize bias associated with model assumptions generally, including the issue of detection, by careful consideration of study design, data collection, and statistical analysis [78]. Using hierarchical occupancy models as an example, [78] showed that, in virtually all realistic cases, accounting for detectability reduces the bias in the estimation of occupancy relative to naïve models even when detectability is heterogeneous. Of course reliable inference depends on sampling methods that produce reasonable odds of detection given presence; no estimator will be particularly helpful when applied to data on populations or species that are “invisible” to sampling [79].

We restricted our quantitative review to a subset of ecological parameters in which imperfect detection may play an important role. But there certainly are other parameters of ecological interest for which inference may be improved by accounting for imperfect detection. For example, most studies of seed dispersal by animals have ignored the implications of seeds that were undetected (but see [80]). Recently, though, methods of varying complexity have been developed to account for the effects of imperfect detection on estimates of seed dispersal and survival [81]–[83]. In general, the current proliferation of detection methods [84]–[86] and statistical techniques for modelling detectability [1], [26], [49] are applicable to a wide range of taxonomic groups, parameters, and experimental scales and present an unprecedented opportunity for ecologists to provide more robust estimation and inference.

## Supporting Information

Dataset S1 Articles included in the literature review. A spreadsheet containing information about papers included in this quantitative literature review, including file names, journals, taxa studied, parameters estimated, and other related values. Detailed metadata are included in the spreadsheet.
(XLSX)

Checklist S1 PRISMA Summary details and locations within the text of specific components of the systematic review conducted in this paper, based on the 2009 PRISMA group guidelines.
(DOCX)
